# Assessment of Oral Health Knowledge, Attitudes, and Behaviours among University Students in the Asir Region—Saudi Arabia: A Cross-Sectional Study

**DOI:** 10.3390/healthcare11233100

**Published:** 2023-12-04

**Authors:** Geetha Kandasamy, Tahani Almeleebia

**Affiliations:** Department of Clinical Pharmacy, College of Pharmacy, King Khalid University, Abha 61421, Saudi Arabia

**Keywords:** knowledge, attitude, behaviour, students, health, non-health, oral health

## Abstract

Background: The purpose of this study is to evaluate the knowledge, attitudes, and behaviours (KAB) of health and non-health-related students at university concerning oral health. Materials and Methods: This cross-sectional study with a 3-month duration in 2023 was conducted amongst King Khalid University (KKU) students. This study used a self-administered, anonymous web-based survey with a simple random sampling strategy as part of its cross-sectional design. The questionnaire had four sections totalling 26 questions to evaluate KAB. A chi-square test was used to evaluate significant differences between categories. Results: Of the 845 respondents who completed the survey, 43.78% were health-related students, while 56.21% were non-health-related students. The completed responses included bacteria causing gingival problems (60.81% vs. 36%), fizzy soft drinks adversely affecting teeth (67.83% vs. 40%), tobacco chewing or smoking possibly causing oral cancer (68.37% vs. 44%), white patches or dental plaque on teeth (61.89% vs. 41.47%), brushing teeth twice a day to improve oral health (81.62% vs. 42.52%), keeping teeth clean (64.86% vs. 41.68%), improper brushing leading to gum disease (70% vs. 40.63%), brushing with fluoridated toothpaste to prevent tooth decay (63% vs. 40%), bleeding gums denoting gum infection (26.98% vs. 21.30%), the importance of teeth similar to any body part (61.62% vs. 37.89%), prioritising teeth as other parts of the body (61.05% vs. 36.21%), brushing teeth twice daily (55.67% vs. 37.05%), and routine dental check-ups (55.40% vs. 14.10%) for health and non-health-related students, respectively (*p* < 0.05). Conclusion: The results of the current study demonstrated less oral health KAB in non-health-related students than in health-related students. Nonetheless, healthcare students provided erroneous information regarding oral health. Therefore, we urge the appropriate authorities to offer health and non-health-related students the best oral health promotion programmes and services, thereby seeking to improve their oral health knowledge while stressing the importance of practising excellent oral hygiene.

## 1. Introduction

People’s general health and overall well-being are greatly influenced by their dental health, which is linked to their awareness of oral health and good oral hygiene practices [1]. Good oral health is when the state of the mouth is free of any disease affecting the oral cavity and surrounding structures. Therefore, a person’s general health and well-being continue to be strongly influenced by oral health. For healthy teeth and gums, maintaining appropriate oral hygiene is as important as maintaining good oral health. These practices also help a person look and feel good [2]. According to the World Health Organization (WHO), approximately 3.5 billion people (35.5% of the world’s population) have permanent dental decay [3]. Periodontitis, a significant cause of tooth loss apart from caries, is a global public health issue from an epidemiological perspective. Oral health knowledge is an essential prerequisite for health-related behaviour, which improves with age. Health-related behaviours are measures individuals take to protect, maintain, and recover their health to prevent disease [4]. Beyond its impact on general health, periodontitis is associated with aspiration pneumonia, cardiovascular disorders, stroke, diabetes, premature birth, and low birth weight [5,6].

Indeed, two sets of behaviours must be continuously practised to maintain good oral hygiene: using dental services (e.g., regular check-ups, oral health promotion, and carefully implemented prevention strategies) and developing self-care routines (e.g., good oral hygiene, reducing sugar, and using fluoride products). Adults should floss their teeth at least once daily, brush them twice daily, and receive routine oral health exams to prevent oral health problems [7].

The WHO reports that despite significant advancements in population-level oral health measures, oral health issues are still not adequately controlled internationally. This state of oral health may be caused by the rapid onset of oral diseases after lifestyle alterations, such as eating a diet high in sugar, drinking non-fluoridated water, and other socio-environmental causes. Oral health is a critical public health concern due to the high incidence and prevalence of oral diseases worldwide. Furthermore, in most industrialised nations, oral disease treatments are the fourth most expensive [8,9].

According to another investigation conducted among university students, the two most susceptible factors affecting the quality of life related to dental health were physical and psychological discomfort [10,11]. Reducing the consumption of cariogenic foods, particularly refined carbohydrates, sugars, and sweets, through a balanced diet is a key component in preventing dental caries [12]. Regular dental exams, at least annually, can identify poor oral hygiene and early carious lesions [13]. Nonetheless, relatively little data are available about Saudi students’ KAB about oral health.

Research on oral health KAB has been carried out among university students in several countries. Peltzer and Pengpid (2014) studied oral health behaviour among undergraduate university students from various specialities in 26 low-, middle-, and high-income countries. This study verified that university students in many African and Asian cultures and the Americas had poor dental attendance and tooth cleaning rates [14]. Moreover, in 2017, a study was conducted by Kumar et al. at a university in Eastern India to examine oral health practices, attitudes, and knowledge among dental and medical students. The researchers found that women had better oral health behaviours and knowledge than men [2]. Other studies evaluated university dental students and compared them with undergraduates from other faculties. The outcomes of these investigations were unsurprising since undergraduate courses for dentistry students previously included education on oral health, compared with non-dental students [15].

Nonetheless, prior research has not concentrated on the KAB of healthcare and non-healthcare students at university. It is crucial to consider that students can help promote health and spread preventive knowledge among their families and the community. We hypothesised that evaluating oral-health-related KAB in health and non-health-related courses impacts university students’ awareness of oral health. The purpose of this study is to evaluate the KAB of health and non-health-related students concerning oral health at King Khalid University (KKU) in the Asir region of Saudi Arabia.

## 2. Methodology

### 2.1. Study Design

The research was conducted for 3 months in 2023 amongst KKU students. This study used a self-administered anonymous web-based survey as part of a cross-sectional design.

### 2.2. Population and Setting

This study was conducted with KKU students in the Asir region of Saudi Arabia and included students from health (e.g., Pharmacy, Medicine, Dental, Nursing, Applied Health Sciences, and Radiology) and non-health departments (English, Engineering, Maths, Computer Science, and Business Management). After understanding the project objectives, KKU students were willing to participate in the study voluntarily. Informed consent was obtained from the student participants. The study excluded those who did not provide consent or who submitted incomplete questionnaires.

### 2.3. Sample Size and Sampling Procedure

The sample size was calculated using the Raosoft online sample size calculator with a specified margin of error of 5% and a confidence level of error of 95% based on the approximate *N* = 2000 as the total number of students at KKU in the Asir region of Saudi Arabia. The target sample size was selected as *n* = 323 to reduce the error in the results and improve the reliability of the study. Initially, 903 students responded, but 58 were excluded due to insufficient data. A simple random sampling strategy was used to select and recruit study participants. The survey was delivered online in various formats, including social media sites, and was created using Google Forms.

### 2.4. Data Collection Tool

A validated questionnaire developed by Selvaraj et al. in 2021 was administered [16]. Our oral health KAB questionnaire’s confirmatory factor analysis confirmed excellent validity in convergent and discriminant terms, along with optimal goodness-of-fit values [17]. The structured questionnaire had four sections, totalling 26 questions. Data on age, gender, and department were gathered in the first section. Eleven questions in the second segment tested the participants’ oral health knowledge. The third section contained eight questions about oral health attitudes. Seven questions on participants’ oral health behaviours were included in the fourth section. The response to each question was yes or no (0 or 1). Students responded to the questionnaire in English. To test the final questionnaire’s language and structure, 25 students from several departments participated in a pilot study. The final results did not include the pilot study results.

### 2.5. Statistical Analysis

Using SPSS version 12, the data were downloaded, coded, submitted, and analysed. Frequencies and percentages were used to describe the results. A chi-square test and logistic regression analysis were used to evaluate significant differences between categories. A *p*-value less than 0.05 was considered significant.

### 2.6. Ethics Approval

KKU’s EMC#2023-2407 Ethical Committee of Scientific Research granted ethical permission. Participants had the option to decline the invitation to participate in the study at any time and did not face consequences for doing so.

## 3. Results

A total of 903 participants responded to our questionnaire survey; 58 responses were excluded due to incomplete data. Among the 845 final respondents, participants from health-related departments (e.g., Pharmacy, Medicine, Applied Health Sciences, Nursing, and Dental) were *n* = 370 (43.78%), while non-health-related department participants (e.g., English, Maths, Computer Science, and Engineering) were *n* = 475 (56.21%). The detailed information is listed in Table 1.

### 3.1. Oral Health Knowledge among Study Participants

Eleven questions were used to assess students’ knowledge about oral health. The detailed information is listed in Table 2.

### 3.2. Oral Health Attitude among Study Participants

Eight questions were used to assess oral health attitudes. The detailed information is listed in Table 3.

### 3.3. Oral Health Behaviour among Study Participants

Seven questions were used to assess oral health behaviour. The detailed information is listed in Table 4.

Age and family members (healthcare employees) were significant (*p* < 0.01) predictors of a good level of knowledge of oral health among non-health-related college students. Smoking and family members (healthcare employees) were significant (*p* < 0.01) predictors of a good level of knowledge of oral health among health-related college students. Gender, study year, and marital status were not significantly associated (*p* > 0.01) with a good level of oral health knowledge amongst non-health-related and health-related college students. The detailed information is listed in Table 5.

Age, smoking, and family members (healthcare employees) were significant predictors (*p* < 0.01) of a good attitude level regarding oral health among non-health-related college students. Smoking and family members (healthcare employees) were significant (*p* < 0.01) predictors of a good attitude level regarding oral health among health-related college students. Gender, study year, and marital status were not significantly associated with the level of oral health among non-health and health-related college students. Detailed information is listed in Table 6.

Family members (healthcare employees) were a significant (*p* < 0.01) predictor (*p* < 0.01) of good oral health amongst non-health-related college students but had a non-significant association with health-related college students. Age, gender, study year, marital status, smoking, and family members (healthcare employees) had a non-significant association (*p* > 0.01) with good oral health behaviour among health-related college students. Age, sex, study year, marital status, and smoking were not significantly associated with good oral health among non-health-related students. Detailed information is listed in Table 7.

## 4. Discussion

Oral hygiene is strongly related to one’s KAB. Knowledge of proper oral hygiene will aid in improving oral hygiene habits. Students of higher educational levels are believed to pay attention to the significance of maintaining proper oral hygiene.

### 4.1. Oral Health Knowledge amongst Study Participants

A person’s general health and well-being continue to be strongly influenced by oral health. Indeed, a comprehensive understanding of and attitudes towards oral health are required for healthy dental practices. When accepted and believed, the knowledge obtained from information turns into action, which becomes a habit. The presence of dental caries is a disease that affects most adults and about 90% of schoolchildren globally; it is especially common in Asia and Latin America [18]. In our study, 60.81% and 36% of health and non-healthcare students, respectively, agreed that bacteria contributed to gingival problems. Most respondents thought that gum bleeding and tooth decay could be caused by bacteria (92%), sugar (89%), and tooth infections (81.3%) [19]. These responses can be primarily attributed to oral ignorance and an excessive intake of refined carbohydrates [20]. Although most students were aware that oral health contributes to aesthetics, highlighting the connection between oral health and the health of the rest of the body could encourage oral health care and oral self-care practices within the student community. This result is consistent with research by Nagesh [21].

In this study, 61.89% and 41.47% of health and non-health students, respectively, agreed that white patches on teeth are called dental plaque. A previous study stated that most students knew of dental calculi (90%; *p* < 0.001). However, most students responded incorrectly regarding the nature of plaque [22]. Notably, previous studies found that self-reporting of daily flossing and annual check-ups accurately predicted the incidences of plaque, calculi, gingivitis, and periodontal destruction [23]. In another study, 45% of the participants agreed that bacterial calculi and plaque are the main causes of gum disease [24]. In this study, 71.08% and 49.68% of health and non-health-related students, respectively, thought that tooth infection causes gum bleeding. Gum bleeding is a sign of gum disease, of which over 50% of students in various categories were aware [25]. The results of this study showed that students in health-related departments had a higher percentage of oral health knowledge than students in non-health-related departments, which is consistent with the findings of earlier studies conducted in Tanzania [26].

Furthermore, most respondents (90%) believed that brushing their teeth prevents caries and gingival problems; nevertheless, 43% brushed their teeth at most once daily [27]. Most students appropriately understood the aetiology, prevention, and periodontal disease symptoms of dental caries, which was consistent with Tanzanian research findings. A larger percentage of children who were adequately informed about the causes, prevention, and symptoms of periodontal disease and dental caries showed that students could retain and recall the learned information as they mature [28]. In this study, 67.83% and 40% of health and non-health-related students, respectively, agreed that fizzy soft drinks affect the teeth adversely. The habit of drinking many soft drinks may result in serious dental problems, such as cavities and degradation of the teeth. Therefore, patients should be informed about the adverse effects of consuming too many soft drinks and advised about preventing dental erosion and caries [29].

Furthermore, 68.3% and 44% of health and non-health students, respectively, were aware that smoking or chewing tobacco can cause mouth cancer. In another study, a large percentage of students in Tanzania and Kenya [30,31] had adequate knowledge of the association between cigarette smoking and mouth cancer. However, according to Ahamed et al., 78% of dentistry students knew what causes oral cancer [32]. This information was probably learnt from the media rather than oral health education classes in universities, which do not teach non-health majors about how smoking causes oral cancer.

### 4.2. Oral Health Attitudes amongst the Study Participants

In this study, 81.82% and 42.52% of health and non-health students, respectively, agreed that brushing their teeth twice daily improves oral hygiene. While most (94%) agreed that brushing reduces periodontal disease, a different study found that women had significantly greater knowledge of the value of flossing in reducing gum problems (*p* < 0.001) [22]. In this study, 64.86% and 41.68% of health and non-health-related students, respectively, considered keeping teeth clean and healthy beneficial to overall health. Research with students from various studies in Saudi Arabia revealed that most participants (94%) of both sexes believed brushing their teeth prevented periodontal disease [33]. Indeed, 91.8% said that good oral hygiene can prevent dental caries and periodontitis. Maintaining good oral health is necessary for general health and quality of life, so practising basic dental hygiene is important. Eliminating dental plaque with routine and appropriate mechanical teeth cleaning is the most efficient way to prevent dental caries or periodontitis [13].

When considering that ‘improper brushing leads to gum disease’, 70% and 40.63% of health and non-health-related students agreed, respectively. Periodontal diseases, including gingivitis and periodontitis, are infectious diseases that result from bacterial infection where the causative bacteria are found in dental plaque [34]. Moreover, in response to ‘sweet retention leads to tooth decay’, 61.86% and 41.05% of health and non-health-related students agreed, respectively. Nevertheless, most stated that cleaning their teeth before bedtime after consuming sugary meals and beverages was enough to keep them healthy [19], thus demonstrating the need to teach patients about reducing sugar intake and implementing further preventive measures, such as regular dental visits and proper oral hygiene habits [35].

Another study stated that almost all respondents (97%) considered consuming sweets to affect oral health, yet approximately 50% admitted to eating sweetened food or drinking daily [27]. When considering ‘brushing with fluoridated toothpaste prevents tooth decay’, 63% and 40% of health-related and non-health-related students agreed, respectively. In another study, 57% of those who used fluoridated toothpaste believed that fluoride-induced teeth strengthening occurs from using fluoridated toothpaste in addition to a healthy diet and regular brushing [36]. Hence, educating students is critical to increasing their understanding of fluoride and its advantages in preventing dental cavities [7]. Most people were aware of smoke damage to teeth and sweet, sticky foods that induce tooth decay [37].

Similar to the findings of our study findings, Wahengbam et al. [37] indicated that more than half of the participants knew that fluoride plays a vital role in the prevention of dental decay, compared to only 29.6% of Saudi students in 2015 and only 18% of Nepali children in 2013 [38]. Although adolescents understand how to brush their teeth, they are less knowledgeable about fluoride toothpaste and dental floss. Therefore, when considering that ‘dentists care only about treatment and not prevention’, 48.10% and 43.36% of health-related and non-health-related students agreed, respectively.

In contrast to our study’s findings, another study revealed that 70% of respondents thought dentists cared mainly about treatment, not prevention [37]. Students studying health-related fields (61.62%) and non-health-related fields (16.84%) agreed that gum bleeding indicates gum infection. According to previous research, more students (55.7%; *p* < 0.001) knew that gum bleeding when brushing is a sign of early inflammation [22]. Although this finding was not statistically significant for all items, a different study found that more students correctly answered questions about preventive behaviours, such as the impact of fluoride on teeth, the impact of sugar retention on dentition, how to prevent tooth decay, the advantages of brushing, and ways to stop gum bleeding [39]. When responding to ‘scaling is harmful to the gums’, 42.16% and 42.94% of health-related and non-health-related students agreed, respectively. Calculi levels are lower with routine scaling and polishing than without [40]. Only a small percentage of students knew that adequate conventional therapy, such as scaling, does not damage the teeth, stating that scaling may cause discolouration, chipping of the outer layer, and tooth scraping [24].

### 4.3. Oral Health Behaviour amongst Study Participants

In our study, 61.05% and 36.21% of health-related and non-health-related students, respectively, gave importance to teeth as much as any other bodily aspect. Most students (88%) knew the link between good dental health and general well-being [41]. According to a study, students studying health-related disciplines were less likely to have sensitive teeth (46.21% vs. 50.31% of students studying unrelated fields). The survey found that only 55.67% and 37.05% of health-related and non-health students, respectively, regularly brushed their teeth twice a day. On the other hand, 86.7% of the students had good attitudes about the importance of brushing their teeth twice a day to prevent oral health issues [42].

Specifically, poor brushing habits are related to tooth decay and gum disease. For instance, 93.6% of respondents said that brushing twice a day improves oral hygiene [19]. In a previous study on dental care habits, 67.4% of the participants reported brushing their teeth twice daily [20]. However, the results were somewhat poorer than those of students majoring in health-related subjects, who were generally considered more informed than those studying in unrelated fields. These health students may have possessed more knowledge due to increased academic exposure in their study programme. Therefore, 40.7% of students brushed for 3–5 min, similar to our study [43]. The percentage of students in non-health departments who brushed their teeth twice a day was lower than Verma et al.’s [25] estimation. The difference may result from societal, cultural, environmental, socioeconomic, and other factors in addition to particular personal characteristics like age, gender, aesthetic concern, and interest. However, our study’s percentage for brushing frequency was greater [44] than in other studies.

Several oral disorders, including those regarded as public health problems like caries and chronic periodontitis, can be treated by effective and regular self-tooth brushing. Most participants in a prior study cleaned their teeth once a day [41]. Specifically, 33.51% and 39.36% of health-related students and non-health-related students, respectively, used their teeth to open the bottle caps of drinks in the study. In our study, toothaches were reported by 45.67% of students who were studying health and 49.47% of students who were not studying health, while gum bleeding during brushing was reported by 44.32% and 50.94% of health-related and non-health-related students, respectively. There was a significant difference between preclinical and clinical students in the issue of bleeding gums [32]. These results corroborated those of Gupta et al. [45] and Al Subait et al. [46], who discovered that more than 50% of individuals linked gum bleeding with gum disease. Nearly 55.40% of health students and 14.10% of non-health students reported routine dental visits as a habit.

According to a previous study, 90% of students said they only went to the dentist when they were in pain and cleaned their teeth once daily with a toothbrush and toothpaste, moving the brush in vertical and horizontal motions [41]. However, Kumar et al. discovered that 89.33% of our dental students and 81% of our medical students recognised the necessity of visiting a dental surgeon [2]. Al Kawas et al. found that compared to 20% of dental students, 46% of medical students waited until they had tooth pain before going to the dentist [47], while Usman et al. discovered that 86% of paramedical students, 75% of medical students, and 69% of dentistry students waited until they had a dental condition before visiting a dentist [48].

Indeed, regular dental exams can prevent oral disease, educate patients, and motivate them to adhere to excellent oral hygiene practices [39]. The current study found that students majoring in health sciences had significantly higher KAB regarding oral health than students majoring in non-health sciences. Therefore, the results of this study may be useful in developing oral health education programmes, preventive measures, and other related activities.

### 4.4. Strengths and Limitations

The study’s strength is that the community will ultimately benefit by using this research as a guide to assist students in both health and non-health disciplines to improve their oral health. Students who study areas unrelated to health may have less understanding due to their educational background, lack of exposure, and interest in science-related subjects. The results may be skewed by over and under-reporting due to social desirability, even while confidentiality is maintained, which is one of the study’s limitations. Only self-reported data were used to determine the study’s results. Consequently, participants may have misinterpreted the questions. Therefore, future research should investigate current difficulties using qualitative research procedures like focus groups or interviews to better understand the KAB among university students. Furthermore, the generalizability of the outcomes can be limited to people with similar settings because of the nature of the study design.

## 5. Conclusions

The current study demonstrated lower KAB in non-health-related students than in health-related students. Nonetheless, healthcare students provided erroneous information regarding oral health. Therefore, we urge the appropriate authorities to offer health and non-health-related students the best oral health promotion programmes and services to improve their knowledge of oral health and the importance of practising excellent oral hygiene. We recommend that comprehensive oral health-related education be included and emphasised in the university curriculum to enhance the KAB with a greater focus on non-health-related students.

## Figures and Tables

**Table 1:** Demographic characteristics of the study participants.

Variables	Total Study Population *n* = 845 (%)	Health Related Colleges *n* = 370 (%)	Non-Health Related Colleges *n* = 475 (%)	*p* Value
**Age**				*p* < 0.001
18–20 years	365 (43.19)	142 (38.37)	223 (46.94)	
21–25 years	297 (35.14)	177 (47.83)	120 (25.26)	
>25 years	183 (21.65)	51 (13.78)	132 (27.78)	
**Gender**				
Male	377 (44.61)	118 (31.89)	259 (54.52)	*p* < 0.001
Female	468 (55.38)	252 (68.10)	216 (45.47)	
**Year of Study**				
First year	92 (10.88)	38 (10.27)	54 (11.36)	*p* = 0.01
Second Year	140 (16.56)	87 23.51)	53 (11.15)	
Third Year	132 (15.62)	61 (16.48)	71 (14.94)	
Fourth Year	153 (18.10)	43 (11.62)	110 (23.15)	
Fifth Year	128 (15.14)	59 (15.94)	69 (14.52)	
Sixth Year	200 (23.66)	82 (22.16)	118 (24.84)	
**Marital Status**				
Single	566 (66.98)	264 (71.35)	302 (63.57)	*p* = 0.01
Married	279 (33.01)	106 (28.64)	173 (36.42)	
**Smoking**				
Yes	420 (49.70)	141 (38.10)	279 (58.73)	*p* < 0.001
No	425 (50.29)	229 (61.89)	196 (41.26)	
**Family Members**				
Healthcare employees	311 (36.80)	159 (42.97)	152 (32)	*p* < 0.001
Non-healthcare employees	534 (63.19)	211 (57.02)	323 (68)	

**Table 2:** Oral health knowledge among health-related and non-health-related Colleges.

Oral Health Knowledge	Health Related Colleges (*n* = 370) *n* (%)		Non-Health Related Colleges (*n* = 475) *n* (%)		*p* Value
	Yes	No	Yes	No	
There are two sets of teeth during a lifetime	268 (72.43)	102 (27.46)	246 (51.78)	229 (48.21)	*p* < 0.001
Tooth infection causes gum bleeding	263 (71.08)	107 (28.91)	236 (49.68)	239 (50.31)	*p* < 0.001
Replacement of missing tooth improves oral hygiene	215 (58.10)	155 (41.89)	162 (34.10)	313 (65.89)	*p* < 0.001
The dental caries of deciduous teeth need not be treated	207 (55.94)	163 (44.05)	187 (39.36)	288 (60.63)	*p* < 0.001
Bacteria is one of the reasons for gingival problems	225 (60.81)	145 (41.62)	171 (36)	304 (64)	*p* < 0.001
Fizzy soft drinks affect the teeth adversely	251 (67.83)	119 (32.16)	190 (40)	285 (60)	*p* = 0.001
Loss of teeth can interfere with speech	218 (58.91)	152 (41.08)	174 (36.63)	301 (63.36)	*p* = 0.05
Irregularly placed teeth can be moved into the correct position by dental treatment	261 (70.54)	109 (29.45)	201 (42.31)	274 (57.68)	*p* < 0.001
Decayed teeth can affect the appearance of a person	225 (60.81)	145 (39.18)	194 (40.84)	281 (59.15)	*p* < 0.001
Tobacco chewing or smoking can cause oral cancer	253 (68.37)	117 (31.62)	209 (44)	266 (56)	*p* < 0.001
White patches on teeth are called dental plaques	229 (61.89)	141 (38.10)	197 (41.47)	278 (58.52)	*p* < 0.001

**Table 3:** Oral health attitudes among health-related and non-health-related colleges.

Oral Health Attitude	Health Related Colleges (*n* = 370) *n* (%)		Non-Health Related Colleges (*n* = 475) *n* (%)		*p* Value
	Yes (%)	No (%)	Yes (%)	No (%)	
Brushing teeth twice a day improves oral health	302 (81.62)	68 (18.37)	202 (42.52)	273 (57.47)	*p* < 0.001
Keeping your teeth clean and healthy is beneficial to your health	240 (64.86)	130 (35.13)	198 (41.68)	277 (58.31)	*p* < 0.001
Improper brushing leads to gum disease	259 (70)	111 (30)	193 (40.63)	282 (59.36)	*p* < 0.001
Sweet retention leads to tooth decay	229 (61.86)	141 (38.10)	195 (41.05)	280 (58.94)	*p* < 0.001
Brushing with fluoridated toothpaste prevents tooth decay	231 (63)	139 (37.56)	190 (40)	285 (60)	*p* < 0.001
Dentists care only about treatment and not prevention	178 (48.10)	192 (51.89)	206 (43.36)	269 (56.63)	*p* = 0.16
Gum bleeding denotes gum infection	228 (61.62)	142 (38.37)	180 (37.89)	295 (62.10)	*p* < 0.001
Scaling is harmful to gums	156 (42.16)	214 (57.83)	204 (42.94)	271 (57.05)	*p* = 0.81

**Table 4:** Oral health behaviour among health-related and non-health-related colleges.

Oral Health Behaviour	Health Related Colleges (*n* = 370) *n* (%)		Non-Health Related Colleges (*n* = 475) *n* (%)		*p* Value
	Yes	No	Yes	No	
I give importance to my teeth as much as any part of my body	290 (61.05)	80 (21.62)	172(36.21)	303 (63.78)	*p* < 0.001
I have sensitive teeth	171 (46.21)	199 (53.78)	239 (50.31)	236 (49.68)	*p* = 0.23
I brush my teeth twice daily	206 (55.67)	164 (44.32)	176 (37.05)	299 (62.94)	*p* < 0.001
I use my teeth to open the cap of bottled drinks	124 (33.51)	246 (66.48)	187 (39.36)	288 (60.63)	*p* = 0.07
I experience toothache while chewing food	169 (45.67)	201 (54.32)	235 (49.47)	240 (50.52)	*p* = 0.27
I have bleeding gums during brushing	164 (44.32)	206 (55.67)	242 (50.94)	233 (49.05)	*p* = 0.06
I have routine dental check-ups	205 (55.40)	165 (44.59)	67 (14.10)	408 (85.89)	*p* < 0.001

**Table 5:** Logistic regression analysis of oral health knowledge among health-related and non-health-related colleges.

Oral Health Knowledge	Health Related Colleges (*n* = 370) *n* (%)					Non-Health Related Colleges (*n* = 475) *n* (%)			
Independent Variables	Groups	Beta Value	OR	95% CI	*p* Value	Beta Value	OR	95% CI	*p* Value
Age	18–20 Years	reference				reference	3.410	1.789–6.499	
	21–25 Years	0.601	1.824	1.080–3.078	0.024	1.227	4.122	2.102–8.083	0.000
	>25 Years	−0.516	0.597	0.286–1.248	0.170	1.416			0.000
Gender	Female	reference				reference	1.212	0.726–2.021	
	Male	0.745	2.107	1.267–3.501	0.004	0.192			0.462
Year of study	1st year	reference				reference	0.543	0.153–1.928	
	2nd year	−0.197	0.821	0.351–1.920	0.649	−0.610	0.527	0.161–1.726	0.345
	3rd year	−0.901	0.406	0.162–1.018	0.055	−0.640	1.791	0.640–5.008	0.290
	4th year	−0.541	0.582	0.218–1.558	0.282	0.583	1.033	0.340–3.138	0.267
	5th year	0.139	1.149	0.450–2.934	0.772	0.033	0.775	0.262–2.290	0.954
	6th year	0.183	1.201	0.487–2.960	0.690	−0.255			0.645
Marital Status	Married	reference				reference	1.288	0.760–2.183	
	Single	0.618	1.854	1.111–3.095	0.018	0.253			0.347
Smoking	No	reference				reference	0.551	0.340–0.893	
	Yes	−0.945	0.389	0.234–0.646	0.000	−0.596			0.015
Family members [Healthcare employees]	No	reference				reference	4.500	2.751–7.359	
	Yes	−0.757	0.469	0.288–0.765	0.002	1.504	0.088		0.000

**Table 6:** Logistic regression analysis of oral health attitudes among health-related and non-health-related colleges.

Oral Health Attitude	Health Related Colleges (*n* = 370) *n* (%)					Non-Health Related Colleges (*n* = 475) *n* (%)			
Independent Variables	Groups	Beta Value	OR	95% CI	*p* Value	Beta Value	OR	95% CI	*p* Value
Age	18–20 Years	reference				reference			0.001
	21–25 Years	0.380	1.462	0.874–2.447	0.148	1.026	2.790	1.536–5.069	0.001
	>25 Years	−0.575	0562	0.270–1.174	0.125	0.953	2.592	1.382–4.861	0.003
Gender	Female	reference				reference			
	Male	0.539	1.714	1.040–2.826	0.035	0.092	1.096	0.673–1.785	0.712
Year of study	1st year	reference				reference			0.198
	2nd year	0.344	1.411	0.613–3.248	0.419	−0.877	0.416	0.133–1.304	0.132
	3rd year	0.099	1.105	0.449–2.718	0.829	−0.464	0.629	0.228–1.733	0.370
	4th year	0.123	1.131	0.427–2.995	0.804	0.199	1.221	0.487–3.057	0.670
	5th year	0.766	2.151	0.847–5.458	0.107	−0.443	0.642	0.233–1.772	0.392
	6th year	0.372	1.451	0.600–3.509	0.409	−0.246	0.782	0.298–2.053	0.617
Marital Status	Married	reference				reference			
	Single	0.685	1.983	1.195–3.291	0.008	0.176	1.192	0.719–1.975	0.496
Smoking	No	reference				reference			
	Yes	−0.883	0.414	0.251–0.680	0.001	−0.693	0.500	0.318–0.788	0.003
Family members [Healthcare employees]	No	reference				reference			
	Yes	−0.716	0.489	0.302–0.791	0.004	1.381	3.980	2.489–6.362	0.000

**Table 7:** Logistic regression analysis of oral health behaviour among health-related and non-health-related colleges.

Oral Health Behaviour	Health Related Colleges (*n* = 370) *n* (%)					Non-Health Related Colleges (*n* = 475) *n* (%)			
Independent Variables	Groups	Beta Value	OR	95% CI	*p* Value	Beta Value	OR	95% CI	*p* Value
Age	18–20 Years	reference			0.749	reference			
	21–25 Years	0.081	1.084	0.627–1.874	0.772	1.089	2.972	1.618–5.462	0.000
	>25 Years	−0.207	0.813	0.365–1.813	0.613	0.608	1.838	0.940–3.590	0.075
Gender	Female	reference				reference			
	Male	1.049	2.854	1.721–4.732	0.000	0.371	1.450	0.874–2.405	0.150
Year of study	1st year	reference			0.096	reference			
	2nd year	0.362	1.436	0.545–3.784	0.464	0.112	1.119	0.349–3.591	0.850
	3rd year	0.690	1.994	0.712–5.584	0.189	0.085	1.089	0.357–3.324	0.881
	4th year	0.338	1.402	0.453–4.337	0.558	0.644	1.905	0.688–5.278	0.215
	5th year	0.859	2.361	0.837–6.661	0.104	0.833	2.301	0.782–6.768	0.130
	6th year	1.265	3.542	1.296–9.683	0.014	0.220	1.246	0.422–3.680	0.691
Marital Status	Married	Reference				reference			
	Single	0.158	1.171	0.667–2.056	0.582	0.315	1.370	0.804–2.335	0.247
Smoking	No	reference				reference			
	Yes	−0.049	0.953	0.563–1.612	0.856	−0.328	0.721	0.449–1.157	0.175
Family members [Healthcare employees]	No	reference				reference			
	Yes	0.408	1.505	0.896–2.5266	0.122	0.676	1.967	1.198–3.227	0.007

## Data Availability

The datasets used and analysed during the current study are available from the corresponding author upon reasonable request.
